# The Interaction between Dietary Selenium Intake and Genetics in Determining Cancer Risk and Outcome

**DOI:** 10.3390/nu12082424

**Published:** 2020-08-12

**Authors:** Shrinidhi Kadkol, Alan M. Diamond

**Affiliations:** Department of Pathology, College of Medicine, University of Illinois, Chicago, IL 60612, USA; skadko2@uic.edu

**Keywords:** selenoprotein, SECIS, selenocysteine, selenium, cancer, polymorphism

## Abstract

There is considerable interest in the trace element selenium as a possible cancer chemopreventive dietary component, but supplementation trials have not indicated a clear benefit. Selenium is a critical component of selenium-containing proteins, or selenoproteins. Members of this protein family contain selenium in the form of selenocysteine. Selenocysteine is encoded by an in-frame UGA codon recognized as a selenocysteine codon by a regulatory element, the selenocysteine insertion sequence (SECIS), in the 3′-untranslated region of selenoprotein mRNAs. Epidemiological studies have implicated several selenoprotein genes in cancer risk or outcome based on associations between allelic variations and disease risk or mortality. These polymorphisms can be found in or near the SECIS or in the selenoprotein coding sequence. These variations both function to control protein synthesis and impact the efficiency of protein synthesis in response to the levels of available selenium. Thus, an individual’s genetic makeup and nutritional intake of selenium may interact to predispose them to acquiring cancer or affect cancer progression to lethality.

## 1. Introduction

Reports emerging in the 1970s indicated an inverse association between the dietary availability of the essential nutrient selenium and cancer incidence, sparking interest in using selenium for chemoprevention. Further evidence for the chemoprotective effects of selenium came from animal models [1,2]. Providing rodents with non-toxic doses of dietary selenium reduced the incidence of induced tumors, and, strikingly, affected several different tissue types and protected against a variety of different carcinogenic insults. These encouraging results led to the Nutritional Prevention of Cancer Trial (NPCT), which tested whether a supplement of 200 μg of selenium in the form of selenized yeast could reduce the incidence of skin cancers among 1312 participants from the Southeast United States previously determined to have an elevated risk for disease recurrence [3]. In this trial, selenium supplementation did not protect from skin cancer recurrence, but secondary analyses indicated that the selenium-supplemented group had a lower incidence of common cancers, including those of colon, lung, and prostate [4]. These results were not the primary endpoints of the study and there were relatively few cancers of these types among the participants, but the findings were considered encouraging enough to initiate other selenium supplementation trials.

Given the NPCT results, prostate cancer seemed a logical target for supplementation studies. Prostate cancer has a high frequency, and it is difficult to predict its course once diagnosed. Additionally, it is difficult to distinguish benign from malignant prostate cancer; thus, a significant percentage of men undergo unnecessary prostatectomies, making prevention an attractive approach to reduce mortality while sparing those diagnosed with prostate cancer from undergoing unwarranted procedures. For these reasons, the National Cancer Institute initiated the Selenium and Vitamin E Cancer Prevention Trial (SELECT) [5], the largest prostate cancer prevention study to date, with over 35,000 North American male participants aged 50 y or older. The study was designed to evaluate whether vitamin E and selenium in the form of selenomethionine, either individually or together, would be more effective than placebo at reducing prostate cancer. After three years, the trial was ended early in 2004 over concerns of elevated prostate cancer incidence in the vitamin E treatment group and a lack of any perceived benefit in the selenium treatment group [6]. Ancillary studies to SELECT have examined whether other cancer type risks were reduced in the selenium supplemented groups but detected no significant benefits [6]. The contrasting results of retrospective studies and supplementation trials may indicate that selenium could prevent cancer through dietary means but not through supplements.

The putative anticancer effects of selenium are likely mediated by selenoproteins as evidence links the expression levels or coding gene polymorphisms of several of these proteins with cancer incidence or outcome [7,8]. Most selenium-containing proteins contain selenium in the form of selenocysteine, often in active sites where the element participates in redox reactions. There are 25 human selenoproteins in this group, many with known enzymatic functions, but their impact on cellular physiology remains to be established [9]. Of note, the levels of approximately half of these selenoproteins are responsive to selenium availability, making these reasonable candidates for the mechanism of selenium chemoprevention indicated by epidemiological studies. In this review, proteins implicated in cancer etiology and whose levels are regulated by selenium will be discussed (Table 1) and the term selenoproteins will be used to refer only to proteins containing selenocysteine.

## 2. Selenoproteins and Their Regulation by Selenium Levels

In general, there are many ways to regulate mRNA translation, but there are some that are unique to RNAs encoding selenocysteine-containing proteins. These transcripts contain an in-frame UGA codon which encodes the amino acid selenocysteine [9]. Distinguishing UGA triplets that direct incorporation of selenocysteine from UGA codons that instead signal translation termination is determined by sequences in the 3′-untranslated region (UTR), which when present result in selenocysteine being added to the growing peptide chain [28,29]. This signal is a stem and loop structure called the selenocysteine insertion sequence (SECIS) [30]. The process of selenocysteine synthesis and insertion requires dedicated translational machinery, which although differing in its specific components, is represented in all Life Kingdoms [31]. In mammals, the process begins with the aminoacylation of a selenocysteine tRNA (tRNA^SerSec^) with serine. The serine is then phosphorylated to phosphoserine, and subsequently selenium is added, resulting in a selenocysteine tRNA [32]. Protein synthesis of selenoprotein-encoding mRNAs then proceeds at the ribosome. If a UGA is encountered and there is a SECIS element in the 3′-UTR, selenocysteine is inserted and translation continues until a functional stop codon signals termination [29,33]. 

Selenoprotein synthesis is also uniquely regulated by selenium levels. Selenium is acquired by cells from the circulation and its intracellular levels influence the availability of specific forms of the tRNA^SerSec^ that favor translation of specific selenoprotein subsets. Most tRNAs contain many modified nucleotides, but tRNA^SerSec^ contains only six [34]. Among these is the modified nucleotide methylcarboxymethyl-5′-uridine (mcm^5^U) 3′ of the anticodon at position 34, which can undergo 2′-O-methylation (mcm^5^Um), with the mcm^5^U and mcm^5^Um containing isoacceptors distinguishable by chromatography. The levels of these isoacceptors differ in various cell types and tissues and are responsive to the selenium concentration in tissue culture media or animal diets [35,36]. When selenium is restricted, there is a shorter half-life and relatively higher level of the isoacceptor with the unmethylated residue mcm^5^U [37]. Using mice genetically engineered to express tRNA^SerSec^ unable to be modified to contain mcm^5^Um, it was determined that the two isoacceptors preferentially participate in the translation of different subsets of selenoproteins. The mcm^5^U-containing isoacceptor participates in the translation of selenoproteins with housekeeping functions while the mcm^5^Um-containing isoacceptor facilitates translation of stress response-associated selenoproteins [38]. Consistent with this, replacing the wildtype tRNA with a mutant tRNA^SerSec^ lacking two modifications, isopentyladenosine at base 37 and U-methylation at position 34, reduced expression of stress-related selenoproteins in a mouse model [38].

Selenium also regulates selenoprotein translation by controlling access of the SECIS binding protein 2 (SBP2) translation factor to the SECIS, a process required for the recognition of UGA triplets as selenocysteine [39]. Selenium deprivation increases production of the DEAD (Asp-Glu-Ala-Asp)box family RNA helicase eIF4a3, which binds to the SECIS element of stress-related selenoprotein transcripts and blocks their interaction with SBP2, a protein essential for selenoprotein translation [40].

## 3. Selenium Transport via Selenoprotein P (SELENOP) and Selenoprotein Synthesis

Reduced levels of several of the 25 human selenoproteins are implicated in cancer etiology [33,41,42]. Thus, it is unsurprising that reduced levels of the selenium carrier protein, which delivers selenium to organs to support selenoprotein synthesis, are also linked to greater cancer risk. Selenium delivery to tissues is accomplished via the 60 kDa selenoprotein P (SELENOP) transport protein [43,44]. SELENOP is synthesized in the liver, where two SECIS elements within the 3′-UTR of SELENOP mRNA are required to incorporate selenium in the form of selenocysteine. SELENOP is uniquely able to incorporate up to 10 selenium atoms during synthesis, which are released upon intracellular digestion of the protein, highlighting its significance as a selenium transporter. After being synthesized in the liver, SELENOP is secreted into the blood plasma, where it is transported into cells by Apolipoprotein E Receptor-2 (ApoER2) mediated endocytosis (reviewed in [45,46]) or, in some tissues, the multi-ligand transport receptor megalin [47]. The transport system used in a particular tissue may influence selenium retention, as some organs, such as the brain and testes, which rely on ApoER2 for uptake, are better able to retain selenium in times of deficiency based on studies of rodents kept on a selenium-restricted diet. Upon entry into the cell, SELENOP is degraded by lysosomes resulting in immediate selenium availability for endogenous selenoprotein production. Due to its role in selenium delivery and bioavailability, SELENOP is a useful marker of selenium levels [48,49]. 

Functional polymorphisms in *SELENOP* were identified by examining SELENOP levels among a diverse group of participants in the Newcastle Heart Project [50]. Volunteers were genotyped at the *SELENOP* locus and provided plasma and lymphocyte samples at baseline, after receiving 100 mg sodium selenite per day for six weeks, and after a 6-week washout period. Two functional polymorphisms within the SELENOP gene were identified in this study, one in the coding region at mRNA position 24731 relative to the start codon resulting in either an alanine or threonine (rs3877899), and one (rs7579) located 14 nucleotides downstream of the in-frame UGA codon resulting in G or A variants. The rs3877899 variants were found to affect the amount of SELENOP at baseline and the rs7579 variants to affect SELENOP concentrations following selenium supplementation. As anticipated given the function of SELENOP in selenium transport, there were associations between the *SELENOP* variants and the levels of several selenoproteins that require selenium for synthesis [50,51]. Among these were selenoproteins associated with protection from oxidative stress, a result supported by a cross-sectional study of oxidative stress markers among New Zealand men [52] and a study of individuals supplemented with selenium-rich Brazil nuts [53]. In addition to affecting the levels of SELENOP, these polymorphisms impacted the distribution between two SELENOP isoforms which differ by approximately 10 kDa [54]. The biological significance of this difference remains to be determined. 

Given the functionality of the SELENOP variations and the impact of selenium availability on selenoprotein synthesis, there have been several studies investigating the associations between *SELENOP* variations and cancer risk. Such associations have been reported for several cancers [7], including those of the colon [10,55,56], breast [57], and prostate [58,59]. In the case of colorectal cancer, the *SELENOP* genotype was reported to interact with selenium status to impact selenoprotein levels and cancer risk [60]. SELENOP variants also interact with polymorphisms in the *SOD2* gene, which encodes another antioxidant protein, MnSOD, to affect prostate cancer risk [61].

## 4. 3′-UTR Polymorphisms in Other Selenoprotein mRNAs Implicated in Cancer Etiology

Polymorphisms within the 3′-UTR of selenoprotein mRNAs have been implicated in cancer risk or mortality and likely impact UGA recoding in response to selenium levels. The impact of 3′-UTR sequences on the translation efficiency of selenoproteins was experimentally established using reporter constructs specially designed to express an mRNA transcript encoding two easily quantifiable reporters, the *lacZ* gene that supports the production of β-galactosidase (β-gal) and the gene for firefly luciferase, separated by a UGA codon [62]. Introduction of the construct by transfection into mammalian cells results in the translation of lacZ with termination at the UGA, but if the construct is altered by the addition of a SECIS element 3′ to the coding sequences, readthrough occurs with the luciferase to -gal ratio reflecting readthrough efficiency and serving as a surrogate for selenoprotein synthesis. These and similar constructs established that the SECIS elements of selenoproteins contain the information for recognizing the UGA as selenocysteine and confirmed that the 3′-UTR sequences of selenoprotein mRNAs influence recoding efficiency in response to selenium availability [2,37,63,64]. A recent manuscript has applied this approach to all 25 human selenoprotein SECIS elements [65]. In addition to being the binding site for the selenocysteine-specific elongation factor SBP2 [39,66], SECIS elements can influence selenoprotein synthesis in response to selenium by binding other regulatory proteins [40], binding to miRNAs [67], or affecting selenoprotein mRNA stability [68,69,70,71,72].

Selenoprotein F (SELENOF) (previously referred to as SEP15 [73]) is an example of a selenoprotein where variations in the 3′-UTR of its mRNA affect expression. SELENOF was originally identified as a human T cell 15 kDa protein that was labeled with ^75^Se and expressed at high levels in several tissues, including the prostate [74,75]. Although the function of SELENOF continues to be investigated, it physically associates with the UDP-glucose:glycoprotein glucosyltransferase (UGTR) in the endoplasmic reticulum (ER) and likely plays an important role in disulfide bond formation and protein quality control in that organelle [76,77,78,79]. SELENOF is unusual as it contains an ER-localization sequence but does not contain an ER-retention signal; retention of SELENOF in the ER is postulated to occur due to its interaction with the ER-resident protein which functions in proper protein folding, UGTR [76]. The *SELENOF* gene is polymorphic in the 3′-UTR and determines the recognition of in-frame UGA codons as the amino acid selenocysteine [74]. The polymorphisms at positions 811 and 1125 (rs5845 and rs5859, respectively) form a haplotype where a C at 811 always corresponds to a G at 1125 and a T at 811 always corresponds to an A at 1125. Using two different specialized reporter constructs, we have shown that these genetic variations are functional and likely contribute to determining the amount of SELENOF protein made as a function of selenium availability [75,80]. 

Genetic data have implicated SELENOF in prostate cancer etiology. A genetic interaction between *SELENOF* and *SELENOP* polymorphisms (rs3877899, rs7579, as discussed above) impacting prostate cancer risk was identified in a cohort of European men [81]. Additionally, a statistically significant association was found between polymorphisms in *SELENOF*, plasma selenium levels, and importantly, prostate cancer mortality [82]. In that study, the 811/1125 polymorphism described above exhibited a trend towards association with prostate cancer-specific mortality with a *p* = 0.10 among a population of self-reported Caucasian men obtained from the Physicians Health Study, where the allele frequency for the at-risk genotype was less than 5%. This is consistent with the low frequency of that SELENOF genotype we previously reported among Caucasians [80]. In contrast, we reported a much higher frequency (31%) of the SELENOF^1125AA^ genotype among African American women [80], and an even higher frequency at 36% was found in a cohort of Chicago-residing African American men [57]. 

African American men have the highest incidence and mortality from prostate cancer as compared to other racial groups in the US, indicated by a roughly two-fold higher incidence rate and a 2.4-fold greater mortality rate compared to Caucasians [83]. Racially distinct regulation of selenoproteins may play a role in these disparities. Notably, African Americans are reported to have lower selenium levels than Caucasians, as determined by examining selenium in plasma and toenails, which indicate long-term selenium status [57,84,85]. This and the frequency of the SELENOF risk genotype among African Americans may contribute to the racial disparities in prostate cancer and perhaps breast cancer. However, the reasons for the racial disparities in prostate cancer are likely multi-factorial, including reduced access to care and other socio-economic factors. In addition, there are a host of biological differences in disease presentation and clinical outcomes which, along with environmental modifiers, likely account for the differences observed between African American and Caucasian men [86,87]. There are also other genetic components; it has been known for decades and confirmed by a recent meta-analysis [88] that individuals with a first-degree relative diagnosed with prostate cancer have a much higher risk of getting the disease.

Glutathione peroxidase 4 (*GPX4*) is another selenoprotein with a 3′-UTR polymorphism implicated in cancer risk or mortality [11,12,55,89]. The relevant polyorphism is a T/C variation at position 718 (rs713041) [13]. GPX4 is an antioxidant enzyme that reduces hydroperoxides within membranes and lipoproteins, acts to prevent the generation of reactive oxygen species (ROS) derived from these peroxides, and, more recently, was found to affect ferroptosis [14,15,16]. The functional role of rs713041 in selenoprotein translation was investigated by quantifying the ability of the different polymorphisms, containing either a C or T at position 718, to support recoding of the UGA codon in the type 1 iodothyronine deiodinase open reading frame when linked to the 3′ end in a reporter construct [17,18]. These constructs were transfected into Caco-2 adenocarcinoma cells, and greater reporter activity was observed in cells expressing the C variant. However, the activity of both reporters decreased significantly when transfected cells were grown in selenium-deficient conditions. Differential binding of an unidentified protein was also reported, with the C-variant binding this protein more tightly [19]. The functionality of these variations was also established in a clinical selenium supplementation trial where volunteers were provided a daily supplement of 100 μg selenium in the form of sodium selenite, and blood samples were analyzed at baseline, at six weeks, and after a washout period [19]. Selenium supplementation increased GPX4 levels and activity, with these GPX4 values falling during the washout phase in *TT* but not *CC* participants. 

The distribution of this polymorphism and establishment of its association with cancer risk was determined in a study genotyping 546 Scottish participants undergoing colonoscopy [17]. Although the C variant exhibited greater reporter activity in cultured cells, the homozygous CC genotype was more prevalent in the germline of adenocarcinoma patients than in patients presenting with adenomatous polyps or in the control group with no evidence of disease. *GPX4* and *SELENOP* polymorphisms also interact to affect colon cancer risk [55], providing additional support for an interaction between selenium status and *GPX4* alleles.

## 5. Selenium and Glutathione Peroxidase 1 Allelic Interactions 

Glutathione peroxidase 1 (GPX1) was identified as an antioxidant protein decades ago and is one of the most studied selenoproteins [20]. It is ubiquitously expressed, resides in the cytoplasm and mitochondria [21,90], and was recently shown to localize to the nucleus in prostate epithelia [57]. GPX1 functions to detoxify hydrogen and lipid peroxides using glutathione as a source for reducing equivalents, yet it is not an essential protein as evidenced by the lack of an overt phenotype in *GPX1* null mice [91]. GPX1 has a low standing in the selenoprotein hierarchy, its levels being among the most sensitive to fluctuations in selenium availability [64,92]. Because of its general role as an antioxidant, the protein has been extensively studied in the context of a variety of diseases, including diabetes, cardiovascular disease, and cancer [93]. 

Allelic variations in the coding sequences of *GPX1* have been extensively investigated as a risk factor for human diseases [7,9,94]. The best investigated polymorphism is a single nucleotide variation affecting codon 198, resulting in either a proline or leucine in the corresponding region of the protein [95]. The consequences of the amino acid at this location remains uncertain, but it is proposed to affect overall structure [22]. Epidemiological studies have revealed inconsistent results regarding the impact of this polymorphism, although most studies have indicated the allele encoding leucine associates with elevated risk [7,96]. The other variation implicated in cancer risk is a variable number of alanine codon repeats, resulting in 5,6, or 7 alanines, in the amino terminus of the protein [23,24]. These trinucleotide repeat variations are thought to impact GPX1 structure and stability [25]. Although neither the codon 198 nor the alanine variations occur in the 3′UTR and, therefore, are unlikely to affect SECIS-controlled UGA recoding, they are likely to contribute to the effects of selenium status on steady state GPX1 levels in a SECIS-independent manner. As an initial screen, 25 immortalized human lymphoblastoid cell lines were genotyped and incubated in standard culture media or media supplemented with 30 nM selenium in the form of sodium selenite [26]. The addition of selenium increased GPX activity 1.3- to 6.9-fold regardless of genotype. To circumvent variables associated with cell line heterogeneity, GPX1 was ectopically expressed from constructs with either a leu or pro encoded by codon 198 and with either 5 or 7 amino-terminal alanine repeats in MCF-7 cells that do not produce detectable GPX1. Cells were then exposed to different amounts of selenium [26]. GPX1 containing 5 N-terminal alanines and a leucine at position 198 had much higher activity and protein levels than the other forms in response to selenium supplementation. Selenium in the media used in these experiments was provided by the 10% serum, resulting in ~25 nM selenium; because maximal GPX activity is not reached, this amount is considered selenium deficiency. These data suggest that GPX1 levels will differ between individuals with the GPX1 protein with five alanines and leucine at position 198 and those with other variants. This speculation has been supported by studies examining selenium status and GPX1 genotype in the prostate [27] and plasma [97]. GPX1 variations also affect the distribution of the protein between the mitochondria and the cytoplasm, which impacts cellular energy metabolism, mitochondrial function, and expression of proteins associated with cancer progression [98,99]. Interactions between the genotypes of *GPX1* and *SOD2*, the gene encoding manganese superoxide dismutase, another antioxidant protein, also impact the regulation of proteins implicated in cancer progression [100].

In addition to *GPX1* polymorphisms, polymorphisms in other selenoprotein genes have been linked to cancer risk when assessed in conjunction with selenium status. Polymorphisms in the genes for two members of the thioredoxin reductase family of selenoproteins (*TXNRD1* and *TXNRD2*) and Selenoprotein K (*SELENOK*) associated with prostate cancer risk only when selenium status was also considered [101]. Neither of these polymorphisms resides in the coding sequence or 3′-UTR, leaving the mechanism behind these associations subject to future studies.

## 6. Conclusions

**Figure 1:**
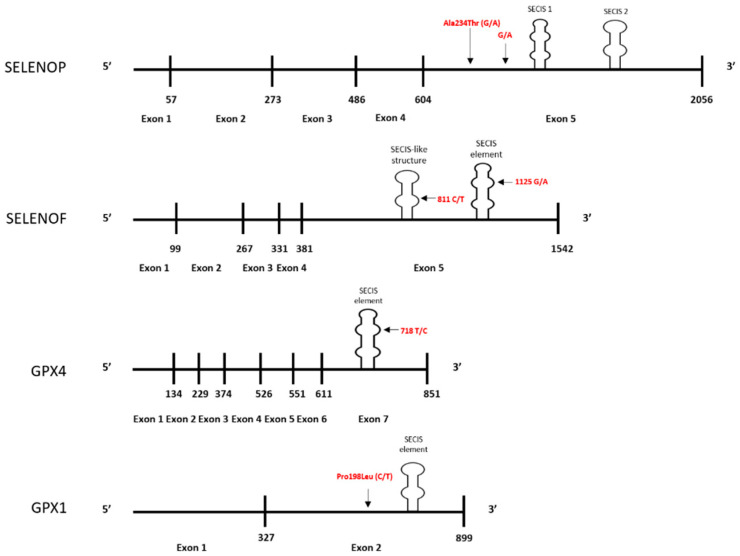
Locations of polymorphisms in selenoprotein genes implicated in cancer and likely impact selenium’s impact on translation. Polymorphism locations for the mRNAs encoded by the *SELENOP*, *SELENOF*, *GPX4*, and *GPX1* genes are shown. Variations in the coding regions and 3′UTR’s of these proteins are indicated in red, and likely impart selenium responsiveness by influencing protein synthesis or selenocysteine incorporation. Vertical lines correspond to exon-exon junctions, which are numbered with the last nucleotide of the preceding exon. Horizontal lines indicate the length of selenoprotein mRNA relative to the other selenoproteins.

Definitive data regarding whether minor alleles of the genes for some selenoproteins can increase cancer risk or mortality have generally been inconsistent. A likely explanation for this is complexity of the interaction between dietary selenium intake and an individual’s genetic profile. Several selenoproteins are implicated in cancer etiology due to studies indicating polymorphisms in at-risk alleles that are also at positions that can influence the levels of the protein, with the risk-associated polymorphisms typically resulting in decreased protein levels when selenium availability is suboptimal for translation. For some proteins, e.g., SELENOF and GPX4, the encoded polymorphisms that are associated with cancer risk results in variations within the 3-UTR of the mRNA that controls UGA recoding and readthrough. In other cases, e.g., the non-synonymous polymorphism and alanine repeats in the coding region of GPX1, the mechanism behind the impact on translation remains less clear. Figure 1 is a schematic summarizing the locations of the polymorphic sites for which data indicates both an association with cancer risk and an influence on the translational regulation by selenium.

A central component to the interaction between selenium and selenoprotein synthesis, and presumably to cancer risk, is SELENOP, which delivers selenium to tissues. Lower SELENOP levels can occur due to sub-optimal intake of selenium or *SELENOP* allelic variations, eventually supporting malignant growth due to the consequential loss of protective selenoproteins. Given that many selenoproteins detoxify reactive oxygen species, the circumstances are even more complicated as variations in proteins in other pathways, such as antioxidant response, DNA repair, and protein transport, may also ultimately determine the benefit or harm attributable to dietary selenium levels. Despite this level of complexity, selenoproteins are still exciting targets for understanding the biology of cancer initiation and progression, as well as the interface between an individual’s genetics and diet.

## Figures and Tables

**Table 1:** Polymorphisms in selenoprotein genes associated with cancer risk or mortality as well as regulation by selenium levels.

Gene	SNP	Location	Association with Selenium/Cancer	Protein Function	Ref
***SELENOP***	rs3877899 (G/A) rs7579 (G/A)	Codon 234 (Ala234Thr) 3′ UTR—SECIS	Reduced breast cancer risk observed for homozygous Thr (rs3877899) carriers. Homozygous AA genotype (rs7579) and interaction of rs3877899 with SOD2 polymorphisms linked to increased prostate cancer risk. Lower SELENOP levels are linked to cancer risk	Transports selenium into cells Antioxidant	[27,28,40,43,44]
***SELENOF***	rs5845 (C/T) rs5859 (G/A)	3′ UTR—position 811 3′ UTR—position 1125	T^811^A^1125^ haplotype associated with prostate cancer specific mortality Haplotypes contribute to determining SELENOF levels made in a selenium-dependent manner	Role in disulfide bond formation/protein quality control Gatekeeper for protein secretion	[57,59,60,61,62,63,64,65]
***GPX4***	rs713041 (T/C)	3′ UTR—position 718	Selenium deficiency causes decreased UGA recoding activity of both variants, while the C variant exhibits increased UGA recoding in selenium-sufficient conditions Clinical supplementation trial - supplementation of 100 ug Se resulted in increased GPX4 levels in participants	Prevents ROS formation by reducing lipid hydroperoxides Structural role in spermatazoa	[10,11,12,13,14,15,16,17,18,19,20]
***GPX1***	rs1050450 (C/T)	Codon 198 (Pro198Leu)	Leu variant is associated with lower GPX1 levels Leu variant with 5 alanine repeat exhibits higher GPX1 induction with Se supplementation compared to other variants. Supplementation of tissue culture media results in increased GPX1 activity and levels in genotype-independent manner	Antioxidant that reduces hydrogen/lipid peroxides	[8,21,22,23,24,25,26,27]
